# Bedside Examination Technique for Taste

**DOI:** 10.21315/mjms2023.30.4.17

**Published:** 2023-08-24

**Authors:** Mohammad Iskandar Sa’uadi, King Peng Lee, Sanihah Abd Halim, Jafri Malin Abdullah

**Affiliations:** 1 Department of Neurosciences, School of Medical Sciences, Universiti Sains Malaysia, Kelantan, Malaysia; 2 Hospital Universiti Sains Malaysia, Universiti Sains Malaysia, Kelantan, Malaysia

**Keywords:** taste pathway, taste disorders, bedside examination technique for taste

## Abstract

Taste disorders are uncommon and frequently unrecognised during neurological and even oral examinations. Nevertheless, understanding taste pathway, its disorders, as well as assessment of taste are crucial as it can reveal various oral, systemic and neurological pathologies that manifest as an alteration of taste. Multiple taste examination techniques have been described in the literature; however, certain techniques are complicated and may not be feasible. This paper describes the adoption of a relatively simple technique for taste assessment that can be performed at the bedside. The bedside detection of taste disorders will allow examiners to assign the patient for more detailed and invasive taste assessments.

## Introduction

Taste is one of the five primary senses that enables humans to identify the odours of different substances. The simultaneous integration of these senses will allow humans to evaluate the nutritious content of food, support oral intake and prevent the ingestion of potentially toxic substances. Generally, there are four basic tastes, namely sweet, bitter, salty and sour. Nevertheless, several researchers have included other tastes like umami (monosodium glutamate [MSG]), disodium guanylate (disodium inosinate), metallic (iron salts) and chalky (calcium salts) (1).

Taste is mediated by specialised epithelial cells that are distributed throughout the oral cavity, oropharynx, larynx and upper third of the oesophagus. Taste buds located on the tongue are scattered on the foliate and circumvallate papillae, each comprising between 50 and 100 taste-receptors cells (TRC) that have a lifespan of approximately 10 days (2). A study on animal models reported that greater taste sensitivity is present near the tip and posterior of the tongue; nevertheless, no segregation of taste qualities has been mapped in humans where TRC are thought to be pluripotent (3).

Initially, the surface of the human tongue was thought to have special areas designated for each taste (sweet, bitter, salty and sour). However, recent findings revealed that tastants are sensed from all parts of the tongue and adjacent structures. Additionally, the afferent nerves to the nucleus of the solitary tract (NST) contain fibres from all types of taste receptors, without any clear localisation of specific types.

### Taste Pathway

Taste pathway begins at taste receptor cells located on the taste buds. Many researchers describe taste buds as a taste-specialised organ that comprises approximately 10,000 ovoid-shaped taste buds, each of which is innervated by approximately 50 nerve fibres. Structurally, taste buds consist of multiple cells which are morphologically distinct, including basal cells, dark cells, light cells and intermediate cells. The half-time of taste cells is about 10 days (4). Furthermore, taste buds are located at several locations within the oral cavity, including the mucosa of the epiglottis, palate, pharynx and on the papillae walls of the tongue. There are three types of papillae, which are circumvallate, fungiform and foliate papillae. Additionally, taste buds respond preferentially, but not solely, to one taste quality.

Taste sensation is carried through the facial nerve (CN VII), glossopharyngeal nerve (CN IX) and vagus nerve (CN X). All taste sensations from the anterior two-thirds of the tongue are carried by the chorda tympani branch of the CN VII, while those from the posterior third of the tongue are carried via the CN IX. Whereas, fibres from pharynx and areas other than the tongue are transported to the brain stem via the CN X. All taste sensations are relayed by respective nerves to the gustatory portion of the NST in the medulla oblongata. From there, second-order neurons ascend to the ipsilateral medial lemniscus and pass directly to the ventral posteromedial nucleus of the thalamus. From the thalamus, the third-order neurons pass to neurons in the anterior insula and frontal operculum in the ipsilateral cerebral cortex. This region is located rostral to the face area of the postcentral gyrus, which is probably the area that mediates the conscious perception of taste and taste discrimination.

### Taste Disorders

Taste alterations are often unrecognised. While quantitative taste disorders are difficult to be detected, complete loss of function or abnormal function is more readily identified. The range of taste disorders includes:

- Dysgeusia: the distorted or altered perception of taste. Dysgeusia may exist as a variety of complaints including metallic, bitter, sour, salty or more rarely, sweet tastes that may be triggered, reduced or unaffected by eating.
- Ageusia or hypogeusia: perceived loss or diminution of function.
- Gustatory hallucinosis or phantogeusia: persistent abnormal taste sensations in the absence of taste stimuli.
- Gustatory agnosia: inability to recognise a taste sensation, even though gustatory processing, language and general intellectual functions are intact.
- Burning mouth syndromes: syndromes of pain with or without taste triggers.

Often, patients who complain of ageusia actually have anosmia because both taste and smell complement each other in producing flavour and full gustatory sensation (3).

In his paper, Bromley (5) listed several disorders that may affect taste sensation. These disorders can be generally classified as disorders of oral cavity, disorders of central nervous system (CNS) or peripheral nervous system, secondary to systemic disorders and disorders from certain medications. Among the range of disorders involving the oral cavity structures that can affect or alter taste perception includes the autoimmune syndrome, lupus erythematosus, infection and ulcerative lesion, local injury, neoplastic lesion, odontogenic diseases, salivary gland abnormalities and paranasal sinus abnormalities.

On the other hand, several conditions and disorders can affect the peripheral nerve, namely: i) infective causes, such as Bell’s palsy, botulism, Guillain-Barre syndrome, Herpes zoster, HIV, Kawasaki disease, otitis media, syphilis and tuberculosis; ii) neoplastic cause for peripheral nerve like glomus tumour, schwannoma, acoustic neuroma and temporal bone cancer; iii) trauma with direct impact on peripheral nerve or its pathway, such as barotrauma, birth trauma, injection of local anaesthesia, middle ear surgery, temporal bone fracture and molar extraction; iv) stroke and v) inflammatory conditions like multiple sclerosis and sarcoidosis.

Once the taste-mediated neuronal information reaches the higher order, another spectrum of disorders may affect the central structures. This mainly involves neurovascular causes, such as stroke, seizures, neoplastic and CNS infection.

Furthermore, systemic disorders and medications can also affect taste. Systemic disorders are secondary to nutritional deficiency, endocrine insufficiency, psychiatric causes (e.g. bulimia), conversion disorder and depression, as well as genetic causes (e.g. familial dysautonomia). It can be addressed using various medications, such as antibiotics, antifungals, anticonvulsants, antidepressants, antihistamines, antihypertensives, antineoplastics, antipsychotics, antiParkinsons, lipid-lowering medications and muscle relaxants.

### Evaluation of Taste

Following the causes of taste disorder discussed above, physicians will normally conduct an assessment of taste as part of the evaluation of CN VII palsy or injury to the CN IX or CN X. Such evaluation usually begins with eliciting a thorough history of the patients, including past and present complaints, previous medical and surgical histories (especially of head and neck procedures), list of medication and nutritional supplement intake, oral intake or habits, and any recent medical or dental management. This can be achieved by asking several questions to the patients, such as:

- Is the taste of food altered or the taste in the mouth changed?
- What is the nature of the taste change: can salt, sour, sweet and bitter be distinguished?

The purpose of such procedure is to explore the onset of symptoms (sudden or gradual) and their progression as well as any precipitating events and/or treatments. Other important symptoms, including smell changes, can also stand as a relevant factor because many patients with smell dysfunction also experience a deficit in taste (6). Moreover, co-morbidities like tobacco, alcohol and exposure to medications or toxins should also be identified. This is followed by a detailed head, neck and oral examination that should include the assessment of cervical lymph nodes, salivary glands, oral cavity, nasal, throat and cranial nerve examination. Whereas, the oral examination should assess the mucosa, teeth and periodontium, oral hygiene and oronasopharyngeal status.

### Methods of Taste Examination

Multiple methods to examine taste sensations have been described in the literature, ranging from the simplest to highly sophisticated (7–10). Generally, these methods can be divided into chemical and electrical tests. Imaging techniques are also included as part of the methods for taste disorder examination as it can reveal abnormalities in oral cavity or the neurological system.

#### Chemical Test

- Three-drop method
- Taste tablets
- Taste strip
- Filter-paper disk (FPD) method

#### Electrical Test

- Electrogustometry
- Gustatory evoked potentials

#### Imaging Test

- Functional MRI (fMRI)
- Positron emission tomography (PET)
- Confocal microscopy
- Photography
- Narrow band imaging (NBI)

This paper describes the procedure involved in the adoption of chemical test for bedside taste examination taste. If the test is deemed abnormal, a multidisciplinary approach can be adopted for further investigation and management.

### Bedside Taste Examination

#### Preparation of Test Items

**Figure 1:**
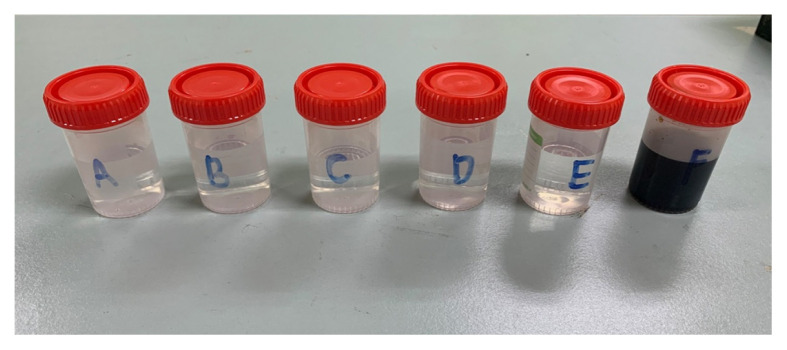
Six bottles of tastant materials (including one bottle of plain water) that were prepared with individual labels

**Figure 2:**
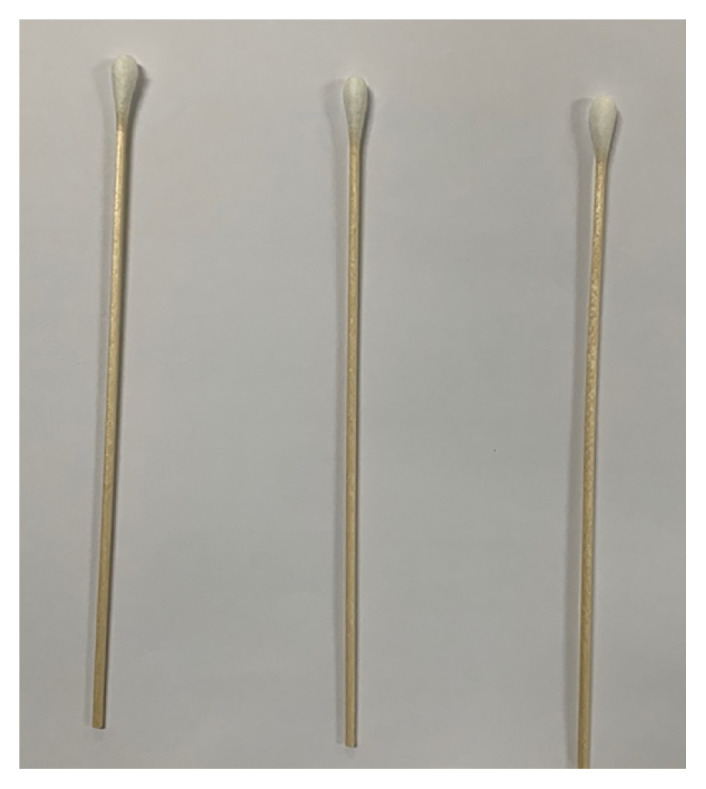
Applicator sticks were used to easily deliver the right amount of tastant materials onto the patient’s tongue

**Figure 3:**
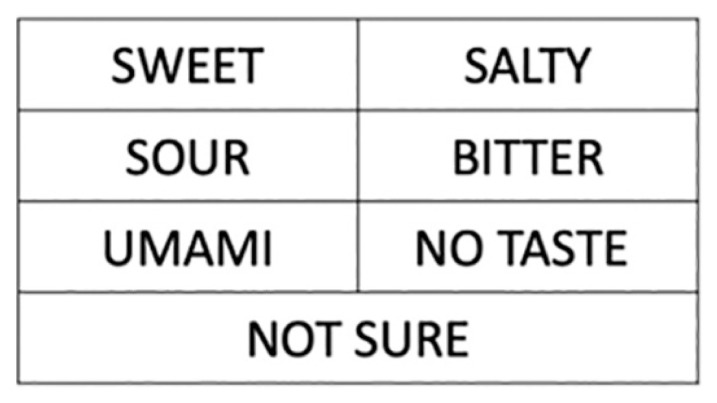
The taste card that was printed for bedside examination

- The preparation of tastants in this study (Table 1) is based on the highest concentration described in Mueller et al. (7). This is because such concentration can be identified by nearly all healthy subjects. Our tastant materials were prepared by the Department of Dieticians, Hospital Universiti Sains Malaysia (HUSM) for accurate measurement and concentration.
  These solutions were prepared with distilled water on the day before for them to equilibrate overnight. They were then portioned into individual coded sample bottles for tasting (Figure 1).
- Plain water (tasteless) was also prepared and randomly placed among the tastant materials.
- Applicator sticks with cotton tips were used to dip the tastants on the patient’s tongue (Figure 2).
- A taste card (Figure 3) was also prepared with the indications of ‘Sweet’, ‘Salty’, ‘Sour’, ‘Bitter’, ‘Umami’, ‘No taste’ and ‘Not sure’.

#### Preparation of Patient

It is important for the patient to not smoke or eat for at least 1 h prior to the test. The patient will be asked to clean the mouth before undergoing the test. Additionally, examiners must ensure that the patient is comfortable and gain verbal consent before the test.

### Examination Procedures

- The examination may begin by explaining the procedures to the patient. For example: “I will examine your head and mouth. I will then place some solutions on your tongue for you to taste. Stick out your tongue and keep it out throughout the test. When you recognise the taste, hold up your hand and point to any indication on the taste card that corresponds to the taste that you perceive.” Since the patient has to keep the tongue protruded and hence will be unable to speak throughout the test, the given instructions must be clear in advance. Once the taste is perceived, the patient may use a signalling system by pointing to words written on the taste card or by making other similar nonverbal responses.
- Conduct a general inspection of the patient. This particularly involves a thorough inspection of the face, head, and neck regions to detect any obvious pathologies.
- Ask the patient to open the mouth. Shed light to inspect any abnormalities within the oral cavity, including teeth, gums and oral mucosa.
- Moistened a cotton tip with one of the tastant materials and use the applicator stick to place it on one half of the tongue (right or left half) in a dabbing manner. Avoid rubbing or swabbing motion. Confine the stimulus to only the tested area of the tongue. Do not allow the patient to retract the tongue because the saliva will diffuse the taste stimulus beyond the test area.
- The tongue must remain protruded throughout the test. Some examiners may prefer to manually hold the patient’s tongue with a gloved hand while holding a piece of gauze to prevent retraction.
- Allow 15 s–20 s for the substance to dissolve and be perceived by the taste receptors. This is because some TRC are metabotropic in nature whereas others are ionotropic (respond immediately upon bound by ligand/ion). Generally sweet, bitter and umami receptors are metabotropic (slower) while salt, sour and some bitter receptors are ionotropic (immediately perceived).
- After 15 s–20 s, ask the patient to respond by indicating the taste perceived on the labelled taste card.
- Repeat steps 4 and 5 with different tastant materials over different halves of the tongue and from anteriorly to posteriorly systematically.

Testing the bitter taste should be done last because it is unpleasant and leaves the most aftertaste. It is seldom necessary or practical to examine taste on the posterior third of the tongue unless a CN IX pathology is suspected.

### Interpretation of Results

Based on this taste examination technique, we can interpret that a patient might have either:

- Normal taste perception.
- Abnormal taste perception. If a patient is unable to respond correctly to the tastant material given, we can further describe the abnormality into dysgeusia or ageusia.
  - Dysgeusia: The patient perceives altered taste sensation. For example, if the patient is given a sweet stimulant, he/she may perceive it as another sensation.
  - Ageusia: The patient is unable to perceive any taste sensations despite multiple tastant materials given.

A video on the bedside taste examination described in this paper is available at https://youtu.be/QMPg8VK_7JM.

## Conclusion

Understanding taste pathway is crucial to localise the level of lesion. It often begins with the taste buds and peripheral nerve until the taste centre. A comprehensive and detailed history followed by systematic examination is paramount in taste evaluation. This paper focuses on a simple bedside examination test that can be performed as part of the screening process to investigate the causes of taste disorders before a patient can be referred for multidisciplinary approaches and proceed with more advanced tests.

## Figures and Tables

**Table 1:** Concentration of substances used according to its basic tastes

Basic Taste	Substance	Concentration
Sweet	Sucrose	0.4 g/mL
Salty	Sodium chloride	0.25 g/mL
Sour	Citric acid	0.3 g/mL
Bitter	Caffeine	0.123 g/250 mL
	Chinine hydrochloride	0.006 g/mL
Umami	MSG	0.25 g/mL
